# Development of a Medication-Related Osteonecrosis of the Jaw Prediction Model Using the FDA Adverse Event Reporting System Database and Machine Learning

**DOI:** 10.3390/ph18030423

**Published:** 2025-03-17

**Authors:** Shinya Toriumi, Komei Shimokawa, Munehiro Yamamoto, Yoshihiro Uesawa

**Affiliations:** 1 Department of Medical Molecular Informatics, Meiji Pharmaceutical University, Kiyose 204-8588, Japan; 2 Department of Pharmacy, National Hospital Organization Kanagawa Hospital, Hadano 257-8585, Japan; shimokawa.komei.gr@mail.hosp.go.jp; 3 Department of Orthopedic Surgery, National Hospital Organization Kanagawa Hospital, Hadano 257-8585, Japan; yamamoto.munehiro.yt@mail.hosp.go.jp

**Keywords:** medication-related osteonecrosis of the jaw (MRONJ), bisphosphonates, epidemiological research, disproportionality analysis, spontaneous report database, FDA Adverse Event Reporting System Database (FAERS), in silico analysis, quantitative structure-activity relationship (QSAR), machine learning, artificial neural network

## Abstract

**Background:** Medication-related osteonecrosis of the jaw (MRONJ) is a rare but serious adverse event. Herein, we conducted a quantitative structure–activity relationship analysis using the U.S. Food and Drug Administration Adverse Drug Reaction Database System (FAERS) and machine learning to construct a drug prediction model for MRONJ induction based solely on chemical structure information. **Methods:** A total of 4815 drugs from FAERS were evaluated, including 70 and 139 MRONJ-positive and MRONJ-negative drugs, respectively, identified based on reporting odds ratios, Fisher’s exact tests, and ≥100 total adverse event reports. Then, we calculated 326 chemical structure descriptors for each drug and compared three supervised learning algorithms (random forest, gradient boosting, and artificial neural networks). We also compared the number of chemical structure descriptors (5, 6, 7, 8, 9, 10, 20, and 30 descriptors). **Results:** We indicated that the MRONJ prediction model using an artificial neural network algorithm and eight descriptors achieved the highest validation receiver operating characteristic curve value of 0.778. Notably, the total polar surface area (ASA_P) was among the top-ranking descriptors, and MRONJ-positive drugs such as bisphosphonates and anticancer drugs showed high values. Our final model demonstrated a balanced accuracy of 0.693 and a specificity of 0.852. **Conclusions:** In this study, our MRONJ-inducing drug prediction model identified drugs with polar surface area properties as potential causes of MRONJ. This study demonstrates a promising approach for predicting MRONJ risk, which could enhance drug safety assessment and streamline drug screening in clinical and preclinical settings.

## 1. Introduction

Medication-related osteonecrosis of the jaw (MRONJ) is a rare adverse event associated with long-term administration of bisphosphonates (BPs) and denosumab [1,2]. However, MRONJ has also been linked to drugs with mechanisms of action distinct from those of bone resorption inhibitors, such as the angiogenesis inhibitors bevacizumab and sunitinib, as well as the immunosuppressants methotrexate and everolimus [1,2,3,4]. This suggests that various drugs may contribute to the development of MRONJ through different pathways. Although MRONJ significantly reduces quality of life, it is recommended to continue treatment with MRONJ-related drugs while implementing strategies to minimize its risk, as the therapeutic benefits of these drugs often outweigh the risks [5,6,7,8]. Therefore, the ability to predict and evaluate the risk of MRONJ in advance would be valuable for managing such adverse events.

Spontaneous reporting systems, which collect data on adverse events in clinical settings over an extended period, play an important role in epidemiological studies, particularly in drug safety evaluations [9,10,11,12,13,14]. A spontaneous reporting system is a drug adverse event database that gathers spontaneous reports from patients, medical professionals, pharmaceutical companies, and other sources. These databases accumulate a huge amount of adverse event reports that are often challenging to obtain at a single institution. In addition, they include data on patients with diverse backgrounds, such as those with renal or hepatic disorders, making a spontaneous reporting system an excellent tool for inductively understanding drug-related adverse events. This reflects not only unique pharmacological and pharmacokinetic characteristics but also prescription and usage conditions [15,16]. In spontaneous reporting system databases, a signal detection approach can be used to identify potential causal relationships between adverse events and drugs, even when such relationships were previously unknown [17,18,19]. Many studies have utilized these databases to explore the association between drugs and adverse events [12,20]. The U.S. Food and Drug Administration (FDA) Adverse Event Reporting System (FAERS) is one of the largest spontaneous reporting databases globally [21].

In recent years, there has been a growing interest in using in silico analysis to evaluate drug toxicity (adverse event evaluation) [22,23]. The physiological activity and physical properties of a drug are typically determined by its chemical structure, which can be analyzed through structural similarity. Quantitative structure–activity relationship (QSAR) analysis is a method that models the relationship between chemical structure and drug efficacy based on this principle [24,25,26,27,28]. This method involves converting the chemical structure of a compound into computationally analyzable features and constructing mathematical models to relate structure to activity. Additionally, machine learning algorithms have been shown to have better performance than traditional logistic regression in predicting binary outcomes [29,30,31]. Because logistic regression analysis is based on the linear relationship between dependent and independent variables, the effectiveness of the model may be reduced significantly for large datasets or variables [32]. However, given that machine learning algorithms are based on nonlinear relationships, they are able to properly recognize and analyze multidimensional and complex features [33]. As a consequence, machine learning models are more effective than logistic regression analysis in large-scale and complex big data analysis [34]. Therefore, by leveraging machine learning to develop an MRONJ-inducing drug prediction model, it becomes possible to assess the risk of MRONJ for various drugs based solely on their chemical structure information. This approach is highly beneficial for screening new compounds and drugs prior to clinical use.

In this study, we integrated data from a drug adverse event database with machine learning techniques to construct an MRONJ-inducing drug prediction model. Initially, drugs associated with MRONJ were extracted from the FAERS drug adverse event database. Subsequently, molecular descriptors representing the structural information of the extracted drugs were calculated, and a classification model for MRONJ-inducing drugs was developed using machine learning. For the MRONJ-induced drug prediction models, we considered three supervised machine learning methods (random forest, gradient boosting, and artificial neural network) and numbers of chemical structure descriptors (5, 6, 7, 8, 9, 10, 20, and 30 descriptors).

## 2. Results

### 2.1. The FAERS Analysis Data Table

The FAERS analysis data table was created by combining information from the FAERS drug table (drug information), Reaction table (adverse event information), Demographic table (basic case information), and Therapy table (treatment period information). Duplicate records were eliminated from the four tables. Following deduplication, the Drug, Reaction, Demographic, and Therapy tables contained 103,252,306, 44,286,680, 14,836,487, and 53,686,946 records, respectively. These tables were merged, and data cleaning procedures were applied to create the FAERS analysis data table. The table contained adverse event data from 12,468,455 records involving 4815 drugs. Among these, 3427 cases (0.027%) were related to MRONJ.

### 2.2. Positive and Negative Drugs for MRONJ

**Figure 1:**
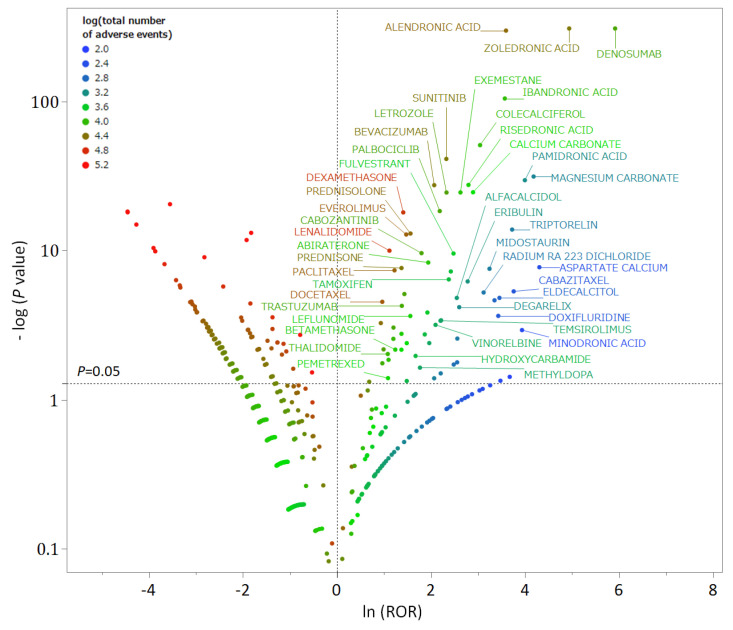
A volcano plot of drugs associated with medication-related osteonecrosis of the jaw (MRONJ). The x-axis represents the natural logarithm of the reporting odds ratios (ln (ROR)), while the y-axis represents the common logarithm of the inverse *p*-value (−log10 [*p*]) from Fisher’s exact test. The dotted line on the y-axis represents *p* = 0.05. The color of the plot represents the total number of adverse events reported for each drug. Drugs associated with MRONJ (MRONJ-positive drugs) are shown in the upper right part of the plot, while drugs not associated with MRONJ (MRONJ-negative drugs) are displayed in the upper left part.

In the FAERS analysis data table, out of 4815 drugs, 70 were identified as MRONJ-positive and 139 as MRONJ-negative (Supplementary Table S1). A volcano plot was generated to visually represent the relationship between the drugs reported in FAERS and MRONJ (Figure 1). Each point on the scatter plot represents a drug, with MRONJ-positive drugs located in the upper right quadrant and MRONJ-negative drugs in the upper left quadrant. The color of each point represents the total number of reported adverse events for each drug, with more red points and fewer blue points indicating higher numbers of adverse events. Among the 70 MRONJ-positive drugs, 11 were classified as malignant tumor drug protein kinase inhibitors (ATC code: L01E), and 8 were drugs affecting bone structure and mineralization (ATC code: M05B), such as BPs (Supplementary Table S1). The number of reported cases of MRONJ was 907 for denosumab, 702 for zoledronic acid, 264 for alendronate, 92 for ibandronate, 65 for sunitinib, and 60 for dexamethasone (Table 1).

### 2.3. QSAR Analysis Data Table

A QSAR Analysis Data Table was created by incorporating 326 chemical structure descriptors for MRONJ-positive and -negative candidate drugs identified from the FAERS analysis data table (Supplementary Table S2). The Simplified Molecular Input Line-Entry System (SMILES) of 70 MRONJ-positive drugs and 139 MRONJ-negative drugs in FAERS was verified, and 326 types of chemical structure descriptors calculated using the Molecular Operating Environment (MOE), a chemical calculation environment, were included. The QSAR Analysis Data Table comprised 60 MRONJ-positive drugs and 108 MRONJ-negative drugs for which descriptors were available. Among the drugs affecting bone structure and mineralization (ATC code: M05B), all six drugs, including BPs, were classified as MRONJ-positive drugs (zoledronic acid, alendronic acid, ibandronic acid, risedronic acid, pamidronic acid, and minodronic acid).

### 2.4. QSAR Analysis Using Machine Learning (Construction of MRONJ-Induced Drug Prediction Model)

In this study, QSAR analysis was conducted to evaluate the machine learning algorithm and the number of chemical structure descriptors to be incorporated into the prediction model. The analysis utilized all chemical structure descriptors for the machine learning algorithms random forest, gradient boosting, and artificial neural network to construct MRONJ-induced drug prediction models (Table 2). Default hyperparameter values of JMP Pro 16.2.0 analysis software were used for the three machine learning algorithms. For random forest, the hyperparameter conditions included 100 trees, 81 terms per branch, and a minimum branch size of 5. Gradient boosting utilized two branches per tree, 48 layers, and a learning rate of 0.02. The artificial neural network employed the Tan H activation function, three layers, and a learning rate of 0.1. Consequently, the area under the receiver operating characteristic curve (AUROC) values in the model validation for random forest, gradient boosting, and artificial neural network using 326 descriptors were 0.726, 0.714, and 0.741, respectively (Table 2). Thus, among the three algorithms, artificial neural network showed the highest prediction accuracy (validation AUROC = 0.741, Table 2).

Furthermore, to enhance computational efficiency and prediction accuracy, we investigated the optimal number of chemical structure descriptors to be incorporated into the artificial neural network prediction model with the highest AUROC (Table 3). Due to the challenge of assessing the importance of each chemical structure descriptor in the artificial neural network prediction model, descriptors with the highest contribution rates from the random forest model were selected (Supplementary Table S3). Using the top 5, 6, 7, 8, 9, 10, 20, and 30 chemical structure descriptors with the highest contribution rates, the validation AUROCs were 0.699, 0.724, 0.719, 0.778, 0.761, 0.748, 0.724, and 0.716, respectively (Table 3). Thus, in this study, the model incorporating the artificial neural network algorithm and the top eight chemical structure descriptors showed the highest predictive accuracy (validation AUROC = 0.778). The validation AUROC value of 0.778 indicates the success of our prediction model in identifying MRONJ-inducing drugs [35].

**Figure 2:**
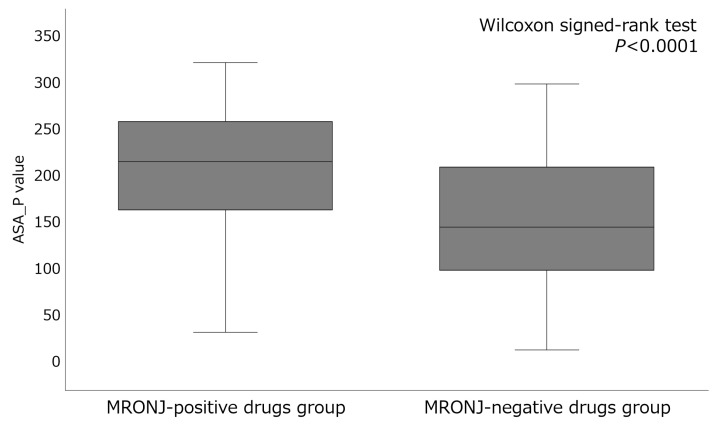
Comparison of the descriptor ASA_P values between MRONJ-positive and -negative drugs.

The eight key descriptors of the top-performing artificial neural network algorithm included ASA_P (total polar surface area), PEOE_VSA_FHYD (fractional hydrophobic dw surface area), PEOE_VSA-5 (total negative 5 dw surface area), h_pavgQ (average total charge), lip_acc (Lipinski Acceptor Count), vsa_acc (VDW acceptor surface area [A**2]), vsa_pol (VDW polar surface area [A**2]), and CASA- (charge-weighted negative surface area) (Table 4). Among these eight descriptors, ASA_P contributed the most. The mean ± standard deviation of ASA_P in the MRONJ-positive and MRONJ-negative drug groups was 220.72 ± 84.95 and 176.09 ± 193.00, respectively, with significant differences (*p* < 0.0001) (Figure 2). Specifically, BPs and anticancer drugs exhibited higher values for ASA_P in the MRONJ-positive drug group (Table 5).

The accuracy rates of the 168 drugs incorporated into the MRONJ prediction model constructed in this study, categorized by drug efficacy group, are presented in Table 6 (top 13 drug classes) and Supplementary Table S4 (all drug classes). Notably, M05B drugs affecting bone structure and mineralization and L04A immunosuppressants achieved accuracy rates exceeding 80%. Conversely, J05A direct acting antivirals and N06A antidepressants had accuracy rates below 0.5.

The predictive model’s performance was assessed by excluding drugs near the cutoff value in the ROC curve to define the applicability domain (Table 7). By setting the applicability domain, the model’s reliability within a specific data range can be determined. Excluding drugs within ±0%, ±10%, and ±20% of the cutoff value resulted in 42, 32, and 17 drugs falling within the applicability domain, respectively, with balanced accuracies of 0.693, 0.750, and 0.800; F values of 0.593, 0.645, and 0.778; and Matthews correlation coefficients of 0.409, 0.488, and 0.618, respectively. Therefore, the performance of the MRONJ predictive model was improved by narrowing the applicability domain.

## 3. Discussion

### 3.1. Analysis of the Adverse Drug Reaction Database FAERS

In this study, we developed a prediction model to classify MRONJ-inducing drugs based solely on their structural information using the FAERS adverse drug reaction database and a machine learning algorithm. To the best of our knowledge, this is the first study to build an MRONJ-inducing drug prediction model utilizing an adverse drug reaction database. Due to the challenge of accurately determining MRONJ risk from the FAERS database, we assessed it using three indicators: reporting odds ratio (ROR), Fisher’s exact test, and the total number of reports for each drug. ROR is widely used for signal detection of adverse events in adverse drug event databases. However, ROR is susceptible to inflation and false signal detection when the number of reports is limited. Therefore, in this study, in addition to ROR, we comprehensively evaluated MRONJ risk by incorporating Fisher’s exact test and the total number of reports.

From the FAERS analysis data table, 80 drugs were identified as MRONJ-positive and 139 as MRONJ-negative. Among the MRONJ-positive drugs, frequently reported drugs included BPs, such as zoledronic acid, alendronic acid, and ibandronic acid; the anti-receptor activator of nuclear factor kappa B ligand (RANKL) antibody denosumab; anticancer drugs like sunitinib, bevacizumab, everolimus, and letrozole; as well as corticosteroids, such as dexamethasone and prednisolone (Table 1). BPs (ATC code: M05B) have a strong affinity for bone hydroxyapatite, inhibit osteoclast activity, reduce bone resorption, and are used to treat osteoporosis and malignant tumors [36]. BPs are closely associated with MRONJ [2]. Monitoring the use of BPs for both malignant tumors and osteoporosis is crucial. Denosumab, an anti-RANKL antibody, has also been linked to MRONJ [37,38]. However, denosumab was not included in the QSAR analysis due to its nature as an antibody preparation. Several protein kinase inhibitors (ATC code: L01E) used in anticancer therapy were also found to be associated with MRONJ, with sunitinib exacerbating MRONJ in renal cell carcinoma [39]. Antiangiogenic drugs also contribute to MRONJ development [40], with varying effects depending on the drug’s mechanism of action [41]. Corticosteroids such as dexamethasone and prednisolone (ATC code: D07A) have been shown to increase the risk of developing MRONJ [42] by delaying wound healing through immunosuppression and altering the oral microbiota, increasing the risk of oral infections and MRONJ [37,43]. Selective estrogen receptor modulators, oral contraceptives, and sex hormone preparations may also influence MRONJ development. Estrogen, a sex hormone, has been shown to impact bone remodeling, potentially affecting jaw bone remodeling [44]. 

This study highlights the potential association between various drugs and MRONJ. While MRONJ is commonly linked to bone resorption inhibitors and antiangiogenic drugs, other medications have also been reported to induce this condition [14,45]. In this study, all drugs registered in the adverse event database were comprehensively examined under the same analysis conditions. The fact that some bone resorption inhibitors and antiangiogenic drugs were detected as MRONJ-positive drugs using this analysis method ensures the reliability of the other detected drugs.

### 3.2. Construction of the MRONJ-Induced Drug Prediction Model

Our goal was to improve the prediction model’s accuracy by comparing three different machine learning algorithms and determining the optimal number of chemical structure descriptors. In the study of three machine learning algorithms, the AUCROCs of random forest, gradient boosting, and artificial neural network were 0.726, 0.714, and 0.741 respectively, with the artificial neural network building the best prediction model (Table 2). While examining the number of chemical structure descriptors, the AUCROCs for each of the prediction models with 5, 6, 7, 8, 9, 10, 20, and 30 descriptors were 0.699, 0.724, 0.719, 0.778, 0.761, 0.748, 0.724 and 0.716, with the prediction models incorporating eight descriptors being the best constructed prediction model (Table 3). Therefore, in this study, an artificial neural network machine learning algorithm and eight chemical structure descriptors were used to build a MRONJ-induced drug prediction model, achieving a validation AUROC of 0.778 (Table 3). To address the challenge of assessing the individual contribution of each descriptor in the artificial neural network, a prediction model was constructed using chemical structure descriptors with a large contribution rate in a random forest. The best MRONJ prediction model consisting of eight descriptors demonstrated a negative predictive value of 0.767, specificity of 0.852, and minimal false negatives (Table 3). Notably, the prediction model exhibited high accuracy rates for drug categories affecting bone structure and mineralization (ATC code: M05B) and immunosuppressants (ATC code: L04A) known to be associated with MRONJ development (Table 6). Our findings suggest that the developed MRONJ-inducing drug prediction model can effectively identify important MRONJ-positive drugs while excluding MRONJ-negative drugs, thereby reducing the risk of overlooking critical medications.

The MRONJ-inducing drug prediction model selected eight molecular descriptors. Many of the descriptors were related to polar surface area, such as ASA_P, PEOE_VSA_FHYD, PEOE_VSA-5, and CASA-; and to van der Waals forces, such as vsa_acc and vsa_pol (Table 4). ASA_P was the most contributing descriptor in this study, and was higher in value in the MRONJ-positive drug group than in the MRONJ-negative drug group (Figure 2). Specifically, BPs and anticancer drugs showed high ASA_P values in the MRONJ-positive drug group (Table 5). BPs are drugs with a high polar surface area that have multiple phosphate and hydroxyl groups, and in this study, the descriptor ASA_P showed a high value. The hydroxyl groups of BPs have a high affinity for bone hydroxyapatite and specifically adsorb to bone cells [46,47]. BPs bound to bone tissue are taken up by osteoclasts, inhibiting their function and reducing bone resorption [48,49]. Additionally, BPs adsorbed to bone cells have been reported to inhibit osteoclast-mediated angiogenesis [50] and affect immune regulation [51]; these mechanisms may influence MRONJ [52]. Similarly, many anticancer drugs designed to target specific molecules have large polar surface areas [53]. Of the 14 anticancer drugs analyzed, 11 protein kinase inhibitors (ATC code: L01E) were confirmed to be MRONJ-positive drugs (Supplementary Table S1). While anticancer drugs have been reported to directly affect osteoclasts and osteoblasts, the underlying mechanism remains to be elucidated [54,55]. Furthermore, the MRONJ prediction model in this study incorporated descriptors related to van der Waals forces, vsa_acc, and vsa_pol. Van der Waals forces play a pivotal role in the binding properties and biological effects of BPs [56]. Particularly, they are thought to facilitate the binding of BPs to the bone and contribute to its stability [57].

The applicability domain of the MRONJ-induced drug prediction model was confirmed to exclude probabilities close to the cutoff value of the ROC curve. By setting the applicability domain within a chemical space, drugs falling within this region can yield reliable prediction outcomes. Consequently, narrowing the application region enhanced the model’s performance.

### 3.3. Limitations

Drug adverse reaction databases such as FAERS have biases and limitations in information collection. FAERS relies on self-reported adverse drug reaction information, which introduces human biases such as reporting bias [58], making it challenging to accurately assess drug risks. Moreover, the detection of false signals of adverse drug reactions may occur when multiple drugs are used [59]. Therefore, in this study, we carefully identified MRONJ-inducing drugs by employing time-series data cleaning methods that consider the characteristics of FAERS and three evaluation indicators (ROR, Fisher’s exact test, and total number of reports) for signal detection.

The quality of a machine learning-based prediction model depends on the quality of the input data [60]. Ideally, reliable data encompassing both positive and negative MRONJ drugs should be included in the learning dataset for QSAR analysis. However, the FAERS data utilized in this study may contain inappropriate reports, leading to limitations in the prediction model’s accuracy. Additionally, due to the rarity of MRONJ, the number of reports in FAERS was limited, resulting in a constrained number of MRONJ-positive drugs in the QSAR analysis dataset. Consequently, the prediction model’s applicability domain in this study may be limited [61,62]. Furthermore, the lack of patient information, such as the genetic background of patients, poses a challenge in explaining individual differences in the onset of adverse events. In future research, Bayesian signal detection methods and cross-validation; standardized scaling methods; and interpretability tools, such as SHAP, can be tested to further optimize our model. Moreover, we plan to validate our model using independent databases (e.g., SIDER and LAREB) or future real-world data to increase its generalizability and clinical utility. Consequently, our findings could be applied to practical strategies for preventing MRONJ in at-risk populations. Moreover, it is essential to determine whether drugs found to be relevant in MRONJ affect the biological pathways involved in osteoclast activity, angiogenesis, and immune regulation to establish a mechanistic association between ASA_P and the MRONJ pathophysiology.

## 4. Materials and Methods

### 4.1. Creation of the FAERS Analysis Data Table

**Figure 3:**
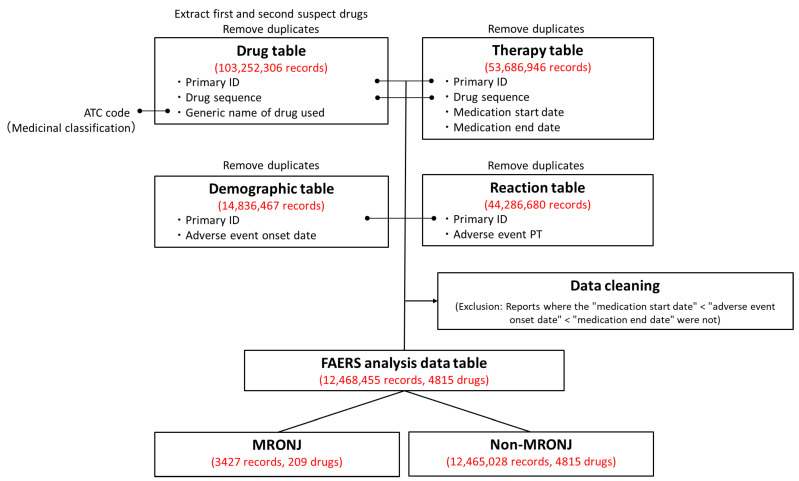
Procedure for creating the U.S. Food and Drug Administration Adverse Drug Reaction Database System (FAERS) Analysis Data Table. Duplicate data were removed from the Drug, Therapy, Demographic, and Reaction tables. Only the “first suspected drug” and “second suspected drug” were extracted from the Drug table. Initially, the Drug and Therapy tables were merged using the Primary ID and Drug sequence. Subsequently, the Demographic and Reaction tables were joined using the Primary ID. To ensure data accuracy, reports that did not adhere to the order of treatment start date, adverse event onset date, and treatment end date were excluded. Out of the 12,468,455 reports in the FAERS analysis data table, 3427 were related to MRONJ.

For the analysis in this study, data reported to FAERS from January 2004 to March 2022 were utilized to create data tables for FAERS analysis (Figure 3). The adverse event data reported to FAERS are stored in seven data tables. In this study, four data tables were utilized: the Drug table (containing drug information), the Reaction table (providing adverse event information), the Demographic table (offering basic case information), and the Therapy table (presenting treatment duration information), with duplicate reports removed [26,27]. The drugs in the Drug table were categorized into first and second suspected drugs and concomitant drugs and interactions, with only the first and second suspected drugs being considered in this study. World Health Organization drug classification ATC codes were assigned to each drug to facilitate drug effect tabulation [63,64]. The Reaction table documented adverse events according to the ICH International Glossary of Pharmaceutical Terms (Medical Dictionary for Regulatory Activities version 25.0; MedDRA ver. 25.0) based on the preferred term [65,66]. In this study, the adverse event “osteonecrosis of the jaw“ in the Reaction table was defined as “medication-related osteonecrosis of the jaw,” with a column added to indicate whether it was MRONJ or not. The Drug and Therapy tables were initially joined using “Primary ID“ and “Drug sequence,” followed by the joining of the Reaction and Demographic tables using “Primary ID.” Additionally, for data cleaning purposes, only data from the Demographic table with adverse event onset dates falling within the Therapy table treatment start and end dates were extracted to create a consistent time-series data table for FAERS analysis.

### 4.2. Examination of the FAERS Analysis Data Tables (Extraction of Positive and Negative MRONJ Drugs)

**Figure 4:**
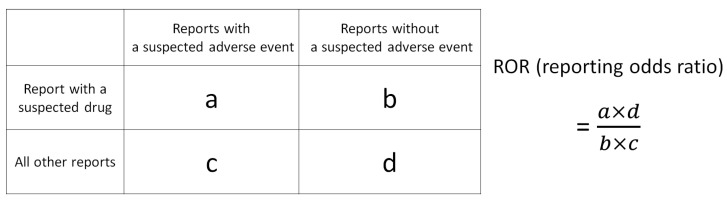
Cross-tabulation and formula used to calculate the ROR for an adverse event. The table is organized with reports for the suspected drug, all other reports, reports with an adverse event, and reports without an adverse event (a–d represent the number of reports).

The drugs in the FAERS Analysis Data Tables were assessed using three indices: the ROR and Fisher’s exact test, along with the total number of reports for each drug. Initially, a 2 × 2 contingency table for MRONJ was created for each of the 4815 drugs in the FAERS analysis data table, and the *p*-values for ROR and Fisher’s exact test were calculated (Figure 4). To stabilize the estimate, a correction was applied by adding 0.5 to all cells (Haldane Anscombe 1/2 correction) [67,68].

ROR is a key indicator in nonproportional analysis methods utilized for detecting adverse drug signals in pharmacovigilance [69]. It offers high sensitivity and low bias, enabling the estimation of the association between the drug and the adverse event [19]. However, classical signal detection indicators such as ROR may overestimate the signal and lead to unstable statistical estimates in cases of low reporting [70,71]. To address this, Eudra Vigilance guidelines recommend a minimum number of reports to ensure a stable signal [72]. In the present study, a threshold of 100 reports (Figure 4; a + b ≥ 100) was set for the total number of reports for each drug (Figure 4; a + b) to prevent the oversight of commonly used drugs [73]. In addition, Fisher’s exact test was used to assess the independence of drugs and MRONJ in the 2 × 2 contingency table in Figure 4. Consequently, the criteria for identifying MRONJ-positive drugs included ROR >1, Fisher’s exact test *p*-values <0.05, and total adverse event reports ≥100, while MRONJ-negative drugs met the criteria of ROR <1, Fisher’s exact test *p*-values <0.05, and total adverse event reports ≥100.

In addition, this study utilized a scatter plot (volcano plot) to visualize the MRONJ-positive and -negative drug candidates from the FAERS analysis data table. Volcano plots, commonly used in bioinformatics to analyze gene expression trends, were employed in this study [73,74,75]. In the volcano plot, the x-axis represents the natural logarithm of the ROR (lnROR), while the y-axis represents the ordinary logarithm [−log (*p*-value)] of Fisher’s exact test *p*-value. The x-axis indicates the risk of MRONJ development when lnROR > 0 (ROR > 1), while the y-axis indicates a −log *p*-value >1.3 (*p* < 0.05), indicating a significant difference in the 2 × 2 contingency table shown in Figure 4. Each point on the plot represents a drug, with the color of the point indicating the total number of adverse event reports (a + b in Figure 4), where drugs with a high number of reports are depicted in red and those with a low number in blue. Only drugs with 100 or more adverse event reports were included in the analysis. Therefore, MRONJ-positive drugs are located in the upper right-hand corner of the plot, while MRONJ-negative drugs are in the upper left-hand corner.

### 4.3. Creation of QSAR Analysis Data Tables (Addition of Chemical Structure Descriptors)

**Figure 5:**
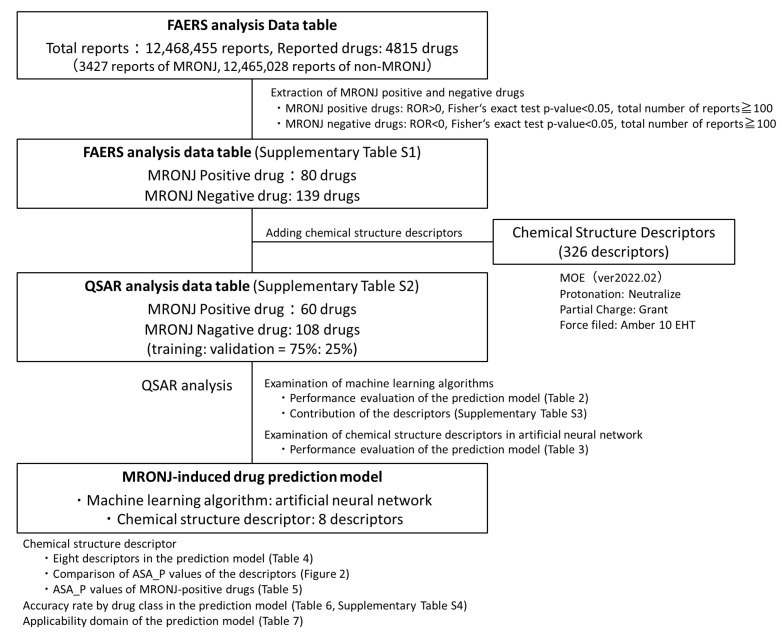
Procedure for building MRONJ-induced drug prediction models. Positive and negative drugs for MRONJ were estimated based on the drugs listed in the FAERS analysis data table. A total of 326 chemical structure descriptions, representing structural features, were added to the positive and negative drugs for MRONJ. In the Quantitative Structure–Activity Relationship (QSAR) analysis, models were constructed and compared using three machine learning algorithms and varying numbers of descriptors. The selection of chemical structure for the artificial neural network was guided by their contribution to the random forest model. The model with the highest performance was checked for accuracy, the significance of the descriptors used, accuracy rates across pharmacological groups of the incorporated drugs, and the applicability domain.

Data tables for the QSAR analysis were created by incorporating chemical structure descriptors for MRONJ-positive and -negative drugs (Figure 5, Supplementary Table S2). The chemical structures of the drugs were obtained from the PubChem compound database in the form of SMILES, a linear representation of molecular structures [76]. Chemical structure descriptors were calculated using the MOE version 2022.02 (Chemical Computing Group, Inc., Montreal, QC, Canada) [77], a specialized chemical computing platform. Prior to descriptor calculations, water molecules and counter ions were eliminated through desalting. Each drug was converted to a three-dimensional structure, assessed for partial charge, and optimized using force field calculations (Amber 10 EHT). A total of 326 chemical structure descriptors were calculated for each drug. Descriptor variables with missing values or perfect collinearity (r^2^ = 1) were excluded. Mixtures, large peptides, bacterial preparations, inorganic compounds, organometallic compounds, and drugs with unspecified names or abbreviations were also removed. Enoxaparin was excluded due to duplication in the dataset. Consequently, the data table for the QSAR analysis comprised 326 chemical structure descriptors for 60 MRONJ-positive and 108 MRONJ-negative drugs. To validate the model, the data table was randomly divided into a 3:1 ratio for training and validation purposes.

### 4.4. QSAR Analysis Using Machine Learning Algorithms (Construction of MRONJ-Induced Drug Prediction Model)

A MRONJ-induced drug prediction model was constructed through QSAR analysis using machine learning algorithms (Figure 5). The algorithms considered were random forests, gradient boosting, and artificial neural networks, all available in the JMP analysis software. Each algorithm has distinct approaches and characteristics. Random forest [78] and gradient boosting [79] are ensemble learning methods that combine several weak learners, such as decision trees. Random forest is known for its stability and improved accuracy through bagging [78], while gradient boosting achieves high prediction performance through sequential error correction by boosting [79]. On the other hand, artificial neural networks consist of multi-layered structures with input, hidden, and output layers containing multiple neurons, enabling them to learn complex nonlinear relationships [80]. In this study, the artificial neural network was constructed using a multilayer perceptron neural network with a back-propagation algorithm for nonlinear regression. Boosting was employed as the ensemble method for the artificial neural network. It is crucial to utilize these algorithms differently due to their unique approaches and characteristics. Although z-score normalization can be beneficial in certain contexts, we did not apply it uniformly in this study because the decision tree-based algorithms (random forest and gradient boosting) used do not strictly require data scaling. Instead, we employed the default preprocessing in our software environment (MOE and JMP). Therefore, three supervised machine learning algorithms were examined and compared by constructing predictive models using default hyperparameters and 326 chemical structure descriptors.

Furthermore, this study investigated the optimal number of chemical structure descriptors in the artificial neural network. It is beneficial to develop predictive models with fewer descriptors for computational efficiency and explainability. Artificial neural networks are effective in capturing nonlinear relationships, but determining the significance of each descriptor in the model can be challenging. On the other hand, random forests utilize decision trees to assess feature importance. In this study, the top chemical structure descriptors with the largest contribution in the random forest model were selected and integrated into the artificial neural network algorithm [81,82]. In addition, we analyzed the descriptors used in constructing the MRONJ predictive model and interpreted the drug characteristics associated with MRONJ.

The predictive performance of the MRONJ-induced drug prediction model was evaluated using metrics such as AUROC, accuracy, precision (positive predictive value), negative predictive value, recall-sensitivity, specificity, balanced accuracy, F1-score, and Matthews correlation coefficient. To mitigate overfitting, we employed a hold-out method with a 3:1 split for training and validation sets in addition to built-in regularization parameters (e.g., limiting the maximum tree depth or minimum branch size).

The applicability domain was assessed by determining the cutoff value on the ROC curve of the artificial neural network prediction model [83]. Defining the scope of application helps to establish the reliable prediction range of the constructed model. In this study, the cutoff value was determined using Youden’s index [84] and normalized to 0.5. Additionally, the model’s performance within the applicability domain was assessed by building a predictive model that excluded drugs with deviations of ±0%, ±10%, and ±20% from the cutoff value.

### 4.5. Statistical Analysis

All analyses were conducted using JMP Pro 16.2.0 (SAS Institute Inc., Cary, NC, USA), and a *p*-value less than 0.05 was considered significant.

## 5. Conclusions

In this study, an MRONJ-induced drug prediction model was constructed using chemical structure information, the FEARS database of drug adverse events, and machine learning. The model, based on an artificial neural network algorithm and eight chemical structure descriptors, identified drugs with polar surface area characteristics as potential contributors to MRONJ. These findings could enhance risk assessment in clinical trials and postmarketing surveillance as well as streamline screening in new drug development. Such prediction models can be integrated into clinical decision support tools to guide personalized drug risk management, highlighting the potential of in silico methodologies in drug surveillance and precision medicine.

## Figures and Tables

**Table 1:** Twenty most frequently reported medication-related osteonecrosis of the jaw (MRONJ)-positive drugs.

Drug Name	Drug Group	Number of MRONJ Reports	ROR	*p*-Value	φ Coefficient
Denosumab	Anti-RANKL antibody	907	373.78	<0.0001	0.136
Zoledronic acid	Bisphosphonates	702	140.70	<0.0001	0.078
Alendronic acid	Bisphosphonate	264	36.69	<0.0001	0.026
Ibandronic acid	Bisphosphonate	92	35.77	<0.0001	0.015
Sunitinib	Anticancer drugs	65	10.30	<0.0001	0.007
Dexamethasone	Corticosteroids	60	4.12	<0.0001	0.003
Cholecalciferol	Vitamin D	55	21.13	<0.0001	0.009
Bevacizumab	Anticancer drugs	51	7.96	<0.0001	0.005
Lenalidomide	Anticancer drugs	47	3.07	<0.0001	0.002
Everolimus	Anticancer drugs	39	4.39	<0.0001	0.003
Letrozole	Anticancer drugs	38	10.34	<0.0001	0.005
Prednisolone	Corticosteroids	36	4.81	<0.0001	0.003
Risedronic acid	Bisphosphonates	33	16.50	<0.0001	0.006
Exemestane	Anticancer drugs	32	13.92	<0.0001	0.005
Palbociclib	Anticancer drugs	31	8.96	<0.0001	0.004
Paclitaxel	Anticancer drugs	29	3.42	<0.0001	0.002
Calcium carbonate	Calcium	28	18.19	<0.0001	0.006
Docetaxel	Anticancer drugs	25	2.64	<0.0001	0.001
Prednisone	Corticosteroids	25	3.97	<0.0001	0.002
Pamidronic acid	Bisphosphonates	22	54.81	<0.0001	0.009

ROR, reporting odds ratio; *p*-value; Fisher’s exact test.

**Table 2:** Examination of the machine learning algorithms.

Machine Learning Algorithms	AUROC of the Training Data	AUROC of the Validation Data	Cutoff Value	Accuracy	Precision/Positive Predictive Value	Negative Predictive Value	Recall/ Sensitivity	Specificity	Balanced Accuracy	F1-Score	Matthews Correlation Coefficient
Random Forest	0.996	0.726	0.533	0.714	0.600	0.778	0.600	0.778	0.689	0.600	0.378
Gradient Boosting	0.956	0.714	0.484	0.714	0.636	0.742	0.467	0.852	0.659	0.538	0.347
Artificial Neural Networks	0.849	0.741	0.526	0.714	0.579	0.826	0.733	0.704	0.719	0.647	0.421

**Table 3:** Examination of the number of chemical structure descriptors in the artificial neural networks.

Number of Chemical Structure Descriptors *	AUROC of the Training Data	AUROC of the Validation Data	Cutoff Value	Accuracy	Precision/Positive Predictive Value	Negative Predictive Value	Recall/ Sensitivity	Specificity	Balanced Accuracy	F1-Score	Matthews Correlation Coefficient
5 Descriptors	0.713	0.699	0.363	0.667	0.600	0.676	0.200	0.926	0.563	0.300	0.186
6 Descriptors	0.837	0.724	0.479	0.714	0.600	0.778	0.600	0.778	0.689	0.600	0.378
7 Descriptors	0.703	0.719	0.554	0.714	0.615	0.759	0.533	0.815	0.674	0.571	0.361
8 Descriptors	0.871	0.778	0.291	0.738	0.667	0.767	0.533	0.852	0.693	0.593	0.409
9 Descriptors	0.871	0.761	0.383	0.714	0.600	0.778	0.600	0.778	0.689	0.600	0.378
10 Descriptors	0.877	0.748	0.265	0.667	0.533	0.741	0.533	0.741	0.637	0.533	0.274
20 Descriptors	0.786	0.724	0.463	0.762	0.778	0.758	0.467	0.926	0.696	0.583	0.458
30 Descriptors	0.777	0.716	0.274	0.738	0.667	0.767	0.533	0.852	0.693	0.593	0.409

* Chemical structure descriptors incorporated into the artificial neural network were used with the top contributions in the random forest.

**Table 4:** Eight chemical structure descriptors contributing to the MRONJ prediction model.

Descriptor	Definition	Number of Branches *
ASA_P	Total polar surface area	7
PEOE_VSA_FHYD	Fractional hydrophobic dw surface area	3
PEOE_VSA-5	Total negative 5 dw surface area	3
h_pavgQ	Total average charge (pH = 7)	3
lip_acc	Lipinski acceptor count	3
vsa_acc	VDW acceptor surface area (A**2)	2
vsa_pol	VDW polar surface area (A**2)	2
CASA-	Charge-weighted negative surface area	2

* Number of branches in the random forest; VDW, van der Waals surface.

**Table 5:** Top 20 MRONJ-positive drugs by descriptor ASA_P values.

Drug Name	ATC Code	Drug Group	ASA_P *
Detirelix	L02BX02	Anticancer drug (hormone-related drugs)	533.2
Triptorelin	L02AE04	Anticancer drug (hormone-related drugs)	509.8
Leuprorelin	L02AE02	Anticancer drug (hormone-related drugs)	412.1
Cefcapene	J01DD17	Antibiotics	322.2
Pamidronic acid	M05BA03	Bisphosphonates	305.2
Alendronic acid	M05BA04	Bisphosphonates	302.7
Pemetrexed	L01BA04	Anticancer drug (metabolic antagonists)	294.9
Docetaxel	L01CD02	Anticancer drug (taxanes)	284.6
Melphalan	L01AA03	Anticancer drug (alkylating agents)	283.3
Bicalutamide	L02BB03	Anticancer drug (hormone-related drugs)	267.5
Epacadostat	L01XX58	Anticancer drug (others)	267.1
Paclitaxel	L01CD01	Anticancer drug (taxanes)	265.3
Zoledronic acid	M05BA08	Bisphosphonates	263.6
Temsirolimus	L01EG01	Anticancer drug (protein kinase inhibitors)	260.5
Allelism	L01EM03	Anticancer drug (protein kinase inhibitors)	259.5
Anastrozole	L02BG03	Anticancer drug (hormone-related drugs)	256.4
Fulvestrant	L02BA03	Anticancer drug (hormone-related drugs)	254.4
Ibandronic acid	M05BA06	Bisphosphonates	252.1
Risedronic acid	M05BA07	Bisphosphonates	244.2
Capecitabine	L01BC06	Anticancer drug (metabolic antagonists)	240.3

* Number of splits in the random forest.

**Table 6:** Accuracy rate by therapeutic class of drugs incorporated into the MRONJ prediction model.

Drug Classes in the ATC Classification	FAERS Analysis Data Table	Classification Results for the MRONJ Prediction Model		
	Number of Drugs (Positive/Negative)	Positive	Negative	Accuracy
L01E Protein kinase inhibitors	14 (11/3)	13	1	0.75
L02B Hormone antagonists and related agents	7 (7/0)	6	1	0.75
L01X Other antineoplastic agents	6 (6/0)	5	1	0.71
M05B Drugs affect bone structure and mineralization	6 (6/0)	6	0	1.00
L04A Immunosuppressants	9 (5/4)	4	5	0.80
A11C Vitamin a and d, incl. combinations of the two	4 (4/0)	4	0	1.00
H02A Corticosteroids for systemic use, plain	4 (4/0)	4	0	1.00
L01C Plant alkaloids and other natural products	4 (4/0)	4	0	1.00
R01A Decongestants and other nasal preparations for topical use	7 (3/4)	5	2	0.56
D07A Corticosteroids, plain	5 (3/2)	5	0	0.43
A07E Intestinal antiinflammatory agents	4 (3/1)	4	0	0.60
C05A Agents for treatment of hemorrhoids and anal fissures for topical use	4 (3/1)	3	1	1.00
S01B Antiinflammatory agents	4 (3/1)	3	1	1.00

**Table 7:** Predictive performance of the MRONJ predictive models for compounds within the application domain.

Applicability Domain	Number of Drugs in Applicability Domain	Accuracy	Precision/Positive Predictive Value	Negative Predictive Value	Recall/Sensitivity	Specificity	Balanced Accuracy	F1-Score	Matthews Correlation Coefficient
Exclusion: Cutoff value 0.5 ± 0 (No exclusion)	42	0.738	0.667	0.767	0.533	0.852	0.693	0.593	0.409
Exclusion: Cutoff value 0.5 ± 0.1 (Applicability: 40–60% exclusion)	32	0.656	0.476	1.000	1.000	0.500	0.750	0.645	0.488
Exclusion: Cutoff value 0.5 ± 0.2 (Applicability: 30–70% exclusion)	17	0.765	0.636	1.000	1.000	0.600	0.800	0.778	0.618

## Data Availability

Data are contained within the article. Data from the FAERS database were downloaded from the website of the U.S. Food and Drug Administration (FDA) (https://www.fda.gov/drugs/drug-approvals-and-databases/fda-adverse-event-reporting-system-faers-database) in 15 June 2022.

## Supplementary Materials

The following supporting information can be downloaded at: https://www.mdpi.com/article/10.3390/ph18030423/s1, Table S1: 70 MRONJ-positive drugs and 139 MRONJ-negative drugs. Table S2: QSAR analysis data table. Table S3: Contribution of chemical structure descriptors in random forests. Table S4: Accuracy rate by therapeutic class of drugs incorporated into the MRONJ prediction model (all drug classes).

## Abbreviations

The following abbreviations are used in this manuscript:

AUROC	Area under the receiver operating characteristic curve
FDA	Food and Drug Administration
MRONJ	Medication-related osteonecrosis of the jaw
QSAR	Quantitative structure–activity relationship
RANKL	Receptor activator of nuclear factor kappa B ligand
ROR	Reporting odds ratio
SMILES	The Simplified Molecular Input Line-Entry System
